# Biennial Variation and Herbivory Affect Essential Oils of *Ipomoea murucoides* and Stomata Density of Neighbor Plants

**DOI:** 10.3390/plants13223124

**Published:** 2024-11-06

**Authors:** José Manuel Sandoval-Moreno, Lilibeth Serrano-Ocampo, Maria Yolanda Rios, María de los Ángeles Ramírez-Cisneros, Alejandro Flores-Palacios, Daniel Tapia-Maruri, Irene de la Concepción Perea-Arango, José de Jesús Arellano-García, Carmen Agglael Vergara-Torres, Susana Valencia-Díaz

**Affiliations:** 1 Centro de Investigación en Biotecnología (CEIB), Universidad Autónoma del Estado de Morelos, Av. Universidad 1001, Col. Chamilpa, Cuernavaca 62209, Mexico; manuel06sm@gmail.com (J.M.S.-M.); iperea@uaem.mx (I.d.l.C.P.-A.); jesus.arellano@uaem.mx (J.d.J.A.-G.); carmen.vergara@uaem.mx (C.A.V.-T.); 2 Facultad de Ciencias Biológicas, Universidad Autónoma del Estado de Morelos, Av. Universidad 1001, Col. Chamilpa, Cuernavaca 62209, Mexico; lilibeth_srn@hotmail.com; 3 Centro de Investigaciones Químicas-IICBA, Universidad Autónoma del Estado de Morelos, Av. Universidad 1001, Col. Chamilpa, Cuernavaca 62209, Mexico; myolanda@uaem.mx (M.Y.R.); angelesrc@uaem.mx (M.d.l.Á.R.-C.); 4 Centro de Investigación en Biodiversidad y Conservación (CIByC), Universidad Autónoma del Estado de Morelos, Av. Universidad 1001, Col. Chamilpa, Cuernavaca 62209, Mexico; alejandro.florez@uaem.mx; 5 Centro de Desarrollo de Productos Bióticos (CEPROBI), Instituto Politécnico Nacional, Carretera Yautepec—Jojutla s/n-km. 85, San Isidro 62739, Mexico; dmaruri@ipn.mx

**Keywords:** allelochemicals, infochemicals, plant–plant communication, volatile organic compounds

## Abstract

Essential oils (EOs) are mixtures of volatile organic compounds that mediate plant interactions and are also appreciated for their biological properties in aromatic plants. However, the study of EOs in wild plants with biological activity has been neglected. *Ipomoea murucoides* is a wild species with allelopathic and insecticide activities; however, the climate factors associated with EOs and their role in intra- and interspecific interactions are still unknown. We investigated the effects of temperature, rain, and solar irradiance for two years on the EOs of *I. murucoides* and documented the effect of herbivory (without, <20%, >20%, and mechanical damage) on their composition. We evaluated the receptivity to possible infochemicals in conspecific and congeneric neighbors to *I*. *murucoides* plants exposed to methyl jasmonate (MeJA), herbivory by *Ogdoecosta biannularis* and without an elicitor. We measured the stomatal density and aperture in the second leaf generation of the neighbor plants. The year and herbivory >20% affected the composition of EOs. Nerolidol could be a biological marker for herbivory. We concluded that herbivory and rain irregularity contribute to EOs changing. The response in the stomatal density in plants not consumed by *I. pauciflora* but near *I*. *murucoides* under MeJA or herbivory gives evidence of interspecific plant–plant communication.

## 1. Introduction

Volatile organic compounds (VOCs) are secondary metabolites of low molecular weight (50–200 Da) that play an essential role in plant defense [1]. Acetaldehyde, methanol, and C_6_ compounds (green leaf volatiles—GLVs) are the first molecules emitted when plants are under abiotic stress [2,3], and benzenoids, mono-, and sesquiterpenes are associated with wounding stress, like herbivory [4]. These molecules participate in the direct and indirect defense of plants and act as infochemicals in plant–plant communication, warning neighbor plants about the risk of herbivory [5,6,7]. Being composed mainly of mono- and sesquiterpenes, as well as phenylpropanoids, essential oils (EOs) are considered a mixture of VOCs primarily obtained by hydrodistillation [8].

EOs are crucial elements in the food, cosmetic, and therapeutic industries [9]; moreover, based on the allomone and kairomone activities of VOCs, EOs are also employed in the agronomical industry [9]. Due to EOs being mainly obtained from aromatic plants [10,11], research their presence in wild plants is neglected. However, some wild species could be a potential source of these molecules because of their interactions and ethnobotanic uses. Screening the variation profiles of EOs in response to biotic and abiotic interactions will allow the detection of associated elicitors. Either biotic or abiotic elicitors, including seasonality and endogenous characteristics, like physiological, genetic, and ontogenic characteristics, modify VOC and particularly EO synthesis [12,13,14]. Moreover, elicitors do not act in isolation; for example, experimental manipulation of temperature and herbivory by *Epirrita autumnata* (Lepidoptera: Geometridae) and *Operophtera brumata* (Lepidoptera: Geometridae) influenced the blend of Eos produced by *Betula nana* L. [15].

The degree of herbivory influences the composition of secondary metabolites [16,17,18]. For example, a fingerprint analysis found that secondary metabolites contained in extracts of *Ipomoea murucoides* Roem & Schult. varied in response to the severity of the herbivory [18]. Actually, the abundance of rare EOs increased in outcrossed plants of *Lepichina floribunda* under simulated herbivory [14]. Thus, the composition of EOs may change when environmental conditions, either biotic or abiotic, become more stressful.

On the other hand, when a plant is being consumed, the mono- and sesquiterpenes are released through the stomata [2] and transported by air, being able to arrive and be adsorbed in the leaf cuticle of a receiver plant (same or different species) [5,19]. These molecules enter the plant through stomata to connect with mesophyll cells and trigger their defense mechanisms [20]. As such, it seems likely that a greater density, size, or number of open stomata will give plants a higher probability of “receiving/hearing” the herbivory alert carried by these molecules. However, in a study with 12 plant species in close spaces, no evidence was found linking the density of stomata and the entry of volatiles [21]. It is likely that because the leaves have a determined growth, changes in the size or density of stomata are only detectable until the next generation of leaves appears after the plant has already been exposed to VOCs.

*Ipomoea murucoides* (Convolvulaceae) is an arboreal species inhabiting central Mexico’s tropical dry forest. Its leaves grow during late spring and are present until early autumn, but, when young, they are subject to a high rate of herbivory due to *Ogdoecosta biannularis* (Boheman) (Coleoptera: Chrysomelidae). Methanolic extracts from the leaves of this species have insecticidal properties against *Spodoptera frugiperda* J. E. Smith. (Lepidoptera: Noctuidae), and hexane extracts from leaves with herbivory (>20%) cause up to 84% mortality of the larvae of this species [18]. On the other hand, bark extracts of *I. murucoides* inhibit the seed germination of the epiphyte *Tillandsia recurvata* (L.) L. (Bromeliaceae) [22,23]. *Ipomoea murucoides* cohabits with *I. pauciflora* M. Martens & Galeotti; still, the leaves of the latter species are eaten in much smaller quantities by *O. biannularis* [24,25]. Because *Ipomoea murucoides* has a rich, non-volatile metabolome, it grows in a highly seasonal system where it faces high pressure from herbivory and has essential biological activities; it is an ideal system to investigate the effect of environmental variations and herbivory on the composition of EOs and the possible conspecific and/or congeneric communication. The questions addressed here are as follows: (a) How does the EO profile of *I*. *murucoides* change in response to climate and herbivory over two consecutive years? and (b) do stomata aperture and density in plants without herbivory change if they grew up near conspecific and congeneric neighbors with herbivory?

## 2. Results

### 2.1. Annual EOs Profile of I. murucoides Considering the Effect of Herbivory

**Figure 1:**
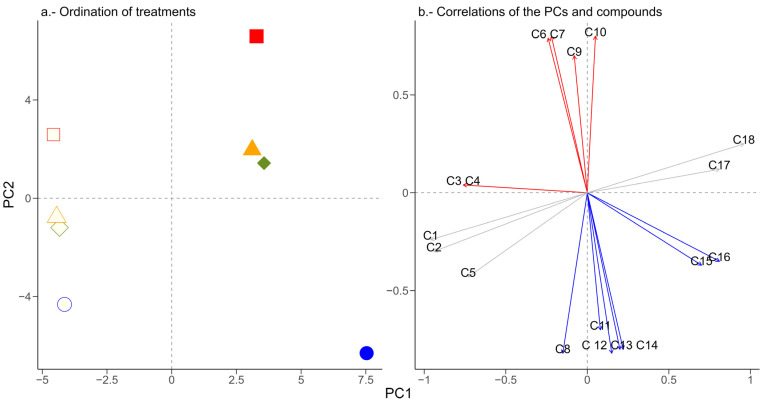
(**a**) Principal component ordination of the VOCs coming from four treatments of herbivory: <20% (rhombs), >20% (squares), mechanical damage (triangles), and without herbivory (circles) during the years 2016 (empty figures) and 2017 (solid figures). In (**b**), we show the correlations of the compounds with the PC. C1 = α-Caryophyllene, 2,6,10-Dodecatrien-1-ol-3,7-11-trimethyl-,(E.E)-,1-Hydroxy-1,7-dymethyl-4-isopropyl-2,7-cyclodecadiene,1-Naphthalenol, 1,2,3,4,4a,7,8,8a-octahydro-1,6-dimethyl-4-(1-methylethyl)-,[1R-(1α,4β,4aβ,8aβ)]-, C2 = phytol, C3 = α-Cubebene, C4 = Cadina-1,4-diene, C5 = α-Farnesene, C6 = (Z)-2-Hexenal, C7 = γ-Elemene, C8 = 1-Naphthalenol, decahydro-4a-methyl-8-methylene-2-(1-methylethyl)-,[1R-(1α,2β,4aβ,8aα)]-, C9 = nerolidol, C10 = τ.Cadinol, C11 = Germacrene D, C12 = τ.-Muurolol, C13 = (*Z*)-3-Hexen-1-ol, C14 = γ-Cadinene, C15 = D25,D18,D7,D19, C16 = α-Muurolene, C17 = D6, and C18 = D3,D2,D1,D8,D4,D12.

From a set of 62 compounds detected in CG-MS, 29 correlated with 2 principal components (PC) (r $\geq \left|0.7\right|$). Both PCs explained 58.57% of the variance; PC1 (34.46% of variance) grouped the herbivory treatments according to the year of the collection, as samples from 2016 were on the left side of the PC1 and those from 2017 were grouped on the right side (Figure 1). PC2 (explained variance = 24.12%) pulls apart the herbivory treatments according to the degree of herbivory. Those EOs that conform to the WH treatment are in the lowest negative axis of PC2. On the other hand, the samples of the herbivory treatment > 20% are in the positive upper side of PC2. The treatments herbivory < 20% and mechanical damage (MD) are grouped toward the center of the PC2 (Figure 1).

**Figure 2:**
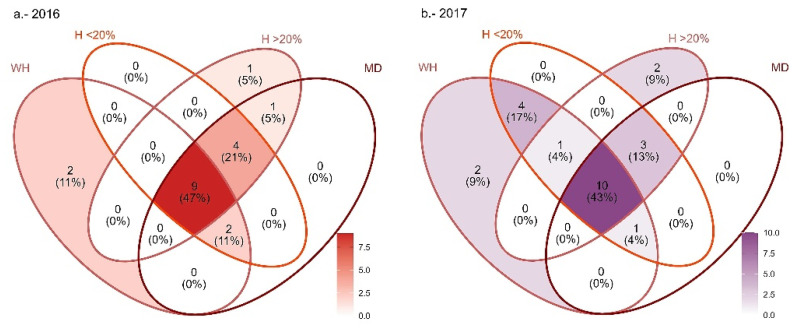
Venn diagrams that represent the distribution of 30 EOs in 4 treatments for (**a**) 2016 and (**b**) 2017. WH (without herbivory), H < 20%, H > 20%, and MD (mechanical damage).

In 2016, 18 chemical compounds correlated with any of 2 principal components, and 23 correlated in 2017 (Figure 2, Table 1). It was observed that both the WH and herbivory > 20% treatments have chemical compounds that define their grouping pattern. In the WH of 2016 and 2017, it was found that the alcohol (*Z*)-3-Hexen-1-ol and the terpene γ-Cadinene (Supplementary Material) were negatively correlated with PC2 (Table 1). Regardless of the collection year, the sesquiterpene alcohol nerolidol (Supplementary Material) was found in herbivory > 20%; this compound correlated positively with PC2. In the same herbivory treatment but only for 2017, α-Cubebene correlated negatively with PC1 (Table 1). There was not a distinctive EO related to the herbivory < 20% and >20% treatments; however, there were compounds present in leaves without damage, including mechanical damage, such is the case of 2-Hexanal and Cadina-1,4-diene, which were both positively correlated with PC1. On the other hand, there were eight chemical compounds in all the herbivory treatments in 2016 (Figure 2a, Table 1); in 2017, there were ten (Figure 2b, Table 1).

### 2.2. Experiment to Determine Changes in Stomata (Intra- and Interspecific)

Regardless of the herbivory treatment, the number of stomata of both species of *Ipomoea* was 28.09 ± 10.01 stomata/0.36 mm^2^ (mean ± standard deviation). When comparing the number of stomata of the two receiver species without considering the herbivory treatment, no significant differences were observed (F_1,46_ = 0.02, P = 0.897). *Ipomoea murucoides* has 28.22 ± 10.31 stomata/0.36 mm^2^, while *I*. *pauciflora* has 27.96 ± 9.90 stomata/0.36 mm^2^.

**Figure 3:**
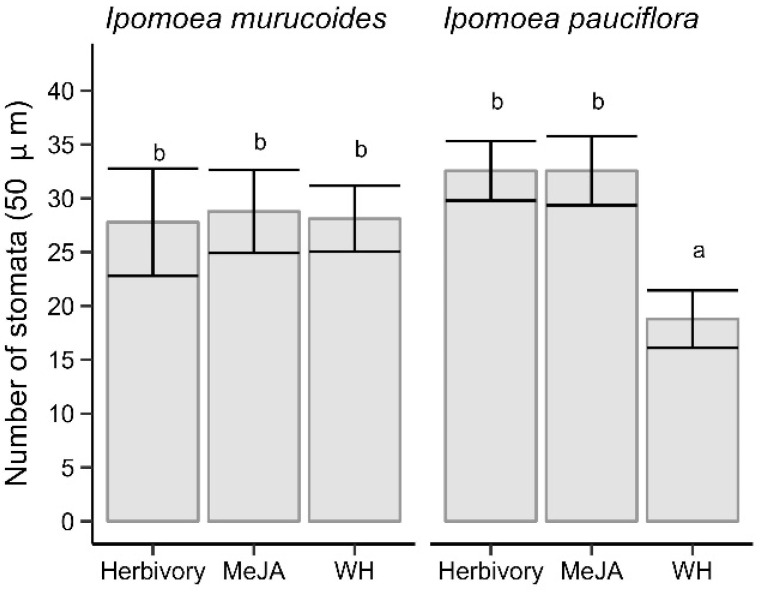
Stomata number in the leaves of two species (*I*. *murucoides* and *I*. *pauciflora*) that were near to an individual of *I*. *murucoides* exposed to herbivory, MeJA, and without herbivory (WH). Dispersion bars are 1SE. Different letters mean significant statistical differences (*p* < 0.05).

**Figure 4:**
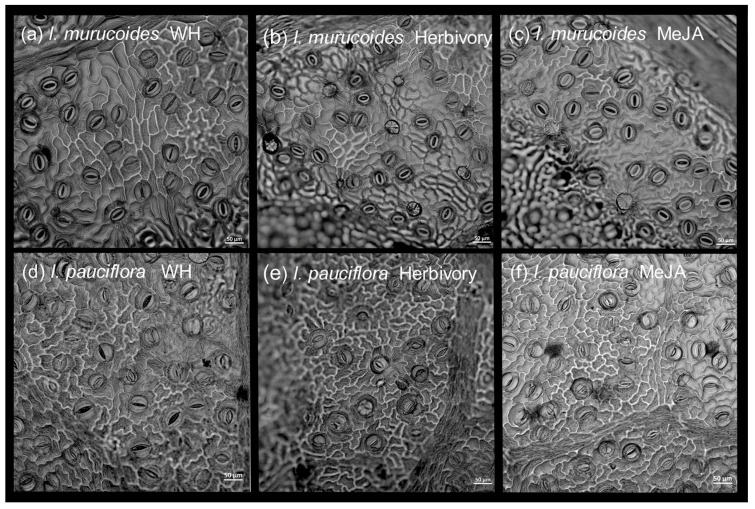
Microphotographs showing the stomata of the abaxial epidermis of leaves of *I. murucoides* and *I. pauciflora* under three treatments. Images were taken with a confocal scanning laser microscopy.

The number of stomata/0.36 mm^2^ in receiver plants close to emitter plants with natural herbivory (30.17 ± 10.65) or sprayed with MeJA (30.67 ± 9.34) was higher (F_2,46_ = 9.02, *p* < 0.001) concerning receiver plants near to emitters without any treatment (23.44 ± 8.81). Moreover, the number of stomata also changes with the interaction of herbivory treatment and the receiver species (F_2,46_ = 10.98, *p* < 0.001). The receiver plants of *I*. *pauciflora* had a statistically minor number of stomata (Tukey test, *p* < 0.05) when they were in the control treatment and near the emitter *I*. *murucoides* (Figure 3 and Figure 4).

Regardless of the species, the percentage of open stomata was 87.5 ± 8.4%. On the other hand, there were no statistical differences in the percentage of open stomata (F_1,1510_ = 0.59, *p* = 0.442) between *I*. *murucoides* (86.8 ± 6.0%), and *I*. *pauciflora* (88.1 ± 10.6%). However, the treatment did affect the number of open stomata (F_1,1510_ = 8.11, *p* < 0.001); MeJA increased the number of open stomata in receiver plants (90.1 ± 3.7%), while the open stomata did not differ between the receiver plants subject to the presence of plants without (89.5 ± 5.3%) or with herbivory (82.6 ± 12.3%). There were no statistical differences between the herbivory treatment × receiver species (F_1,1510_ = 0.73. *p* = 0.483).

## 3. Discussion

Knowledge of the biotic and abiotic factors that affect the plant metabolome allows us to understand the ecological interactions of wild plants and the importance of detecting EOs with economic importance. This is the first study that screened *Ipomoea murucoides* EOs and explored the effect of climate and herbivory on these molecules over two consecutive years. Moreover, we suggest possible congeneric communication between *I*. *murucoides* and *I*. *pauciflora* through changes in the total stomata and the proportion of opened stomata of putative receiver plants of *Ipomoea pauciflora*.

### 3.1. Annual EOs Profile of I. murucoides Considering the Effect of Herbivory

In this study, the year-to-year variation was the main factor causing the ordering in EOs composition. This result is challenging to understand because changes in the metabolomes of plants growing in natural conditions could result from multiple factors [13,43,44] that were not considered in the present study.

Although we considered the effect of the volume of rain on EO composition, the rain regularity (number of days without rain in a period) before and during the month of leaf collection (May and June) may be a determinant in EO variation. The precipitation was more abundant and irregular in 2016 than in 2017. 2016 had 26 days without rain in 61 days of the rainy season, while 2017 had only 18 days of drought in the same period (CONAGUA, https://smn.conagua.gob.mx/es/climatologia/informacion-climatologica/informacion-estadistica-climatologica, accessed on 1 October 2021). Previous studies evidence that the rain volume and regularity affect the production of the aldehyde 2-hexanal by *Punica granatum* L. [43] and terpenoids (α-phellandrene, O-Cymene, γ-terpinene, or β-Caryophylene) in *Thymus vulgaris* L. [44], as possibly occurred in the present study.

EO biosynthesis also depends on ontogeny, genetic, and morphological factors [13,45]. In perennial plants, such as *I*. *murucoides*, it is feasible to distinguish chemical diversity throughout their phenological stages or ages from the seasonal effect because they live for several years, and each stage of development has particular physiological processes [46]. Although we sampled the same individuals in both years and used the same protocol to obtain EOs, the trees’ aging could be responsible for this chemical variation. As the trees became older (2016 to 2017), they exhibited different EO compositions (see the Supplementary Material for chromatograms). In addition, *I*. *murucoides* is a deciduous species that, at the study site, sprouts in late spring, and its leaves are excised in winter; differences between years may be attributable to asynchronous phenology (i.e., earlier sprouting in a year), which in turn could impact the population size and/or feeding habits of the principal herbivore (*Ogdoecosta biannularis*) and, consequently, influence the composition of VOCs in *I*. *murucoides*. Previous evidence points out that phenology affects foliage quality in *Eucalyptus*. Expanding leaves of *Eucalyptus* had different concentrations of secondary metabolites (i.e., sideroxylonals) from those that were fully extended; this variation impacts the abundance of its herbivores [47], and changes in metabolome depend on the herbivory density [21,23].

Secondary metabolites are synthesized constitutively or as a resistance mechanism against herbivory [48]; some are pre-formed molecules stored in specialized structures and have a protective function against stressors [2,3], for example, by diminishing the damage caused by reactive oxygen species on the cell membrane [49]. Regardless of the herbivory treatment or the year, leaves of *I*. *murucoides* have three constitutive sesquiterpenes (α-Cadinene, 7(11)-selinen-4α-ol, and τ-muurolol). To have found constitutive secondary metabolites in *I*. *murucoides* that display diverse biological activities is just a sign that some of these molecules participate in essential functions, like photosynthesis. For example, the diterpene phytol is the main constituent of chlorophyll [50], and it is also in the bark of *I. murucoides* [22]. Moreover, as plants are sessile organisms, they must permanently mediate their interactions with the environment through secondary metabolites. Some studies report that γ-Cadinene, also found here, displays functions as a kairomone [51] in mixtures with other antifungal [52], antifeedant, and insecticidal essential oils [53].

On the other hand, herbivory activates different pathways to synthesize sesquiterpenes and green leaf volatiles (GLVs) [54], which are not emitted synchronically. GLVs are the first to be released because of their smaller molecular mass and high vapor pressure [2], and terpenes are emitted even hours or days after damage [55]. It is possible that GLVs were less represented in our study because they were emitted immediately after herbivory; this would have taken place when the insect activity began (10–11 h), and the leaves of *I*. *murucoides* were collected during the first hours of the day, when the emission of sesquiterpenes persists.

The EO components were grouped according to the herbivory treatment, but the main distinction was between the herbivory > 20% and without herbivory treatments. Along with herbivory > 20%, two sesquiterpenes were correlated with PC2, namely nerolidol (positively), which can be considered a biomarker of stress, and α-cubenene (negatively). The antiherbivore properties of nerolidol have been reported before [56], for example, in *Camellia sinensis*, nerolidol and the phytohormones jasmonic and salicylic acids increase when plants are consumed by herbivores [57]. The presence of α-cubenene has been reported previously as a constitutive VOC in leaves of *Quecurs ilex* without herbivory [55].

In both years, the leaves without herbivory or mechanical damage have γ-cadinene and the GLV (*Z*)-3-Hexen-1-ol, which correlated negatively with PC2. These sesquiterpenes have already been associated as constitutive VOCs emitted by *Quercus ilex* [55]; meanwhile, (*Z*)-3-hexen-1-ol is one of the most abundant VOCs in the atmosphere, and its emission depends on abiotic or biotic stressors [58]. Leaves of sampled trees of *I*. *murucoides* have different degrees of herbivory; although (*Z*)-3-Hexen-1-ol was found in wounded leaves, it is not uncommon to see it distally as a systemic response in undamaged leaves, because this molecule plays roles in direct and indirect plant defense and acts as a warning molecule in plant–plant communication [59].

The EO compositions between the herbivory < 20% and mechanical damage treatments were similar. This similarity may be caused by the small number of perforations made in the leaves of *I*. *murucoides* in the mechanical damage treatment. The few differences between them would be caused by compounds elicited by herbivore-associated molecular patterns (HAMPs) found in oral secretions, saliva, or fluids that come along with oviposition [60]. With our results, we can conclude that *Ipomoea murucoides* plants have a pattern of chemical response to herbivory; if the damage is low, then the pattern is like that caused by mechanical damage, but when the damage is more than 20%, the EO composition differs from mechanical damage and the control without herbivory.

### 3.2. Experiment to Determine Changes in Stomata (Intra- and Interspecific)

Because the essential oil components of *Ipomoea murucoides* changed in response to herbivory, it could probably exert an infochemical activity. *Ipomoea murucoides* and *I*. *pauciflora* are sympatric species from the tropical dry forest of central Mexico. Every summer, the chrysomelid *Ogdoecosta biannularis* consumes leaves of both *I*. *murucoides* [24] and *I*. *pauciflora*. However, the frequency of the insect is greater on *I*. *murucoides* [25], even with toxic compounds, such as pyrrolizidine-type alkaloids [61]. This may evidence the adaptation by the insect to this kind of chemical; in addition, our results showed that the number of stomata of *I*. *murucoides* was independent of MeJA or herbivory, which would also suggest adaptation of *I*. *murucoides* through tolerance mechanisms to the consumption by *O*. *biannularis*. However, the number of stomata in *I*. *pauciflora* increased in the MeJA and herbivory treatments, representing that the number of stomata is a plastic character that enables the plant’s “eavesdropping” in the presence of herbivory. This interspecific plant communication has already been reported [62,63]. When *Artemisia tridentata*, under herbivory or experimental clipping, emits MeJA, this triggers the defense of *Nicotiana attenuata* by inducing the synthesis of the enzyme polyphenol oxidase (PPO); based on the level of herbivory [53], PPO also participates in the synthesis of GLVs [54].

Here, we conclude that the essential oil content of *I*. *murucoides* differed in both years, probably due to the environmental characteristics within each year (i.e., rain periodicity). The high degree of herbivory (greater than 20%) also exerted changes in EOs; hence, some of these molecules may play a defensive role against abiotic and biotic stress, such as in the case of nerolidol. Moreover, we provide evidence that the stomata change in density when interspecific neighboring occurs between two *Ipomoea* species. However, it is desirable that future research takes into account the molecular and physiological variables that could affect changes in EO composition.

## 4. Materials and Methods

### 4.1. Study Area and Species

All plant materials (i.e., leaves and seeds) of *I. murucoides*, and *I*. *pauciflora* were collected from the tropical dry forest located on the hill “Cerro de la Cruz” (18°57′22.2″ W, 99°06′50.2″ N, 1495 m a.s.l.) located in San Andrés de la Cal, Tepoztlan, Morelos, Mexico. This area belongs to the protected area designated as the ‘Biological Corridor “Chichinautzin”’ (http://www.dof.gob.mx/nota_detalle.php?codigo=4793772&fecha=30/11/1988, accessed on 1 February 2024). As San Andrés de la Cal comprises communal lands, the area’s representative permitted us to carry out this investigation. The climate is highly seasonal; the tropical dry forest is the primary type of vegetation, composed of deciduous trees of low stature [64]. The generation of leaves occurs at the end of the dry and at the beginning of the rainy season (late May–September); the mean summer precipitation is 511 ± 21.82 mm, and the mean summer temperature is 20.65 ± 0.65 °C [65]. Regarding the summer solar radiation average, it is 35,295.72 wh/m^2^ (https://smn.conagua.gob.mx/es/climatologia/informacion-climatologica/informacion-estadistica-climatologica, accessed on 31 October 2024). The climate is semi-warm-subhumid. The soil type in the valley is luvic phaeozem; but in hills there are leptosol, characterized by abundant limestones [66]. The most abundant tree species are *Conzattia multiflora* (B.L. Rob.) Standl. (Fabaceae), *Bursera fagaroides* (Kunth) Engl., and *Bursera glabrifolia* (Kunth) Engl. (Burseraceae), *Sapium macrocarpum* Müll. Arg. (Euphorbiaceae), *Ipomoea murucoides*, and *I. pauciflora* [67].

*Ipomoea murucoides* (3–8 m tall) and *I. pauciflora* (<8 m tall) have mature leaves in summer exclusively; they are similar species whose fruits are capsules with four seeds. The leaves of *I*. *murucoides* are lanceolate, while those of *I*. *pauciflora* are heart-shaped; their white corolla is bell-shaped, but the calyx of *I*. *murucoides* is pubescent while that of *I*. *pauciflora* is glabrous [68]. Like other Convolvulaceae, both produce a great diversity of glycosidic resins [69,70]. Vouchers of both species were collected, herborized, and deposited in the herbarium HUMO-Universidad Autonoma del Estado de Morelos and identified by M. Sc. Gabriel Flores Franco (Vouchers No 31059 and 3106, for *I*. *pauciflora* and *I*. *murucoides*, respectively).

The chrysomelid *Ogdoecosta biannularis* is the main folivore of *I*. *murucoides* [24]_,_ and to a lesser extent, it consumes leaves of *I*. *pauciflora* [25]. *Ogdoecosta biannularis* reaches its highest population density in the summer (June–September); the development from egg to adult occurs in approximately 30 days, and the females lay between 40 and 100 eggs [24].

### 4.2. Annual EOs Profile of I. murucoides Considering the Effect of Herbivory

In June 2016 and 2017, in the study area, we collected the leaves from ten randomly chosen *I. murucoides* individuals, including marked individuals from prior research [18,22]. According to the previous methodology [18], we collected leaves with experimental mechanical damage (MD), without herbivory (WH), with herbivory < 20%, and with herbivory > 20%. MD was achieved by perforating each leaf three times with a commercial perforator (diameter = 6.25 mm). Due to the wild conditions of leaf collection and to decrease the probability that collected leaves shared vascular bundles, we ensured that leaves came from separate branches. To avoid the loss of volatiles from the rising temperature during the day, leaf collection was carried out before sunrise (5:00–6:00 h). The collected leaves were hermetically bagged in plastic Ziplock bags, frozen, and transported to the Laboratorio de Botanica Estructural (CEIB-UAEM), where samples were stored at −72 °C in an ultra-freezer (REVCO) until EOs were obtained.

To obtain EOs, 140 g of frozen leaves from each herbivory treatment were weighed (ADAM electronic balance, model Nimbus). Frozen samples were crushed and placed in a ball flask (500 mL) to which 300 mL of distilled water were added. Essential oils were obtained by hydrodistillation in a Clevenger trap (constant temperature = 225 °C/4 h). To separate water traces from the collected essential oils, they were put un Eppendorf tubes and centrifugated at 1000 rpm/5 min. Final samples of both years were analyzed using gas chromatography–mass spectrometry (GC-MS) at the Centro de Investigaciones Químicas (CIQ-UAEM) with the following conditions: samples were dissolved in dichloromethane (5 mg/mL) and analyzed in a gas chromatograph (Agilent 6890) coupled with a sQ 5973 mass detector. The column used was an HP-5MS of 30 m length × 0.25 mm i.d. × 0.25 µm film thickness, with He as the carrier gas. The injection volume was 1 µL in splitless mode, and elution started at 50 °C, increasing 2 °C/min to 285 °C, where it was maintained for 20 min. Data were processed in the ChemStation version 4 (Agilent) software, and compounds were identified by comparison with the NIST.v14 database (Agilent, Santa Clara, CA, USA). Additional confirmation was carried out with the NIST online version (https://www.nist.gov/srd/nist-special-database-14, accessed on 2 November 2024). A standard mix of alkanes (Sigma-Aldrich product name—40147-U-) was analyzed in the same instrument using the same method as for the samples in order to calculate the linear retention index with the Vandendool–Kovatz method.

The temperature, precipitation, and solar irradiation data were obtained from the Automatic Meteorological Station of Tepoztlan (CONAGUA, unpublished data). Data correspond to early summer (May–June 2016 and 2017) when the leaves of *I. murucoides* were collected. Both years’ average mean temperatures, precipitations, and solar irradiations were 24.49 ± 0.21 °C, 162.43 ± 37.18 mm, and 35,295.78 ± 1081.40 W, respectively.

### 4.3. Experiment to Determine Changes in Stomata (Intra- and Interspecific)

#### 4.3.1. Plant and Insect Obtention

Plants of both *Ipomoea* species were obtained through the germination of seeds collected at the study site in February 2018. Before germination, the dispersion structures (setae) were retired from each seed. The seeds were disinfected with sodium hypochlorite (Cloralex^®^) at 0.25%/3 min. The seeds were washed three times with distilled water to eliminate any remaining chlorine. Subsequently, five seeds of each species were placed in Petri dishes (seven Petri dishes for *I. murucoides* and three for *I. pauciflora*) using filter paper with 6 mL of distilled water as a substrate. Petri dishes were sealed with Parafilm to avoid moisture loss and placed in an environmental chamber (Lumistell ICP 55, Celaya, Mexico) at 25 °C, 12 h light/12 h darkness.

Seedling acclimation began after five days of germination had occurred. Seedlings were placed in plastic containers with 16 g of cotton wetted with 250 mL of distilled water. Containers were sealed with a layer of plastic film (Kirkland) and set in an environmental chamber (Lumistell ICP 55, México) at the same seed germination conditions. Ten days later, seedlings were individualized in plastic cups with lava rock (50%), sterile soil from the study site (25%), and peat moss (25%). Then, 10 mL of water were added to each cup, which stayed in the same environmental conditions for 15 more days. Finally, plants were transplanted in plastic bags (15 cm) employing the same substrate and placed in a greenhouse (mean temperature = 22.67 °C and relative humidity = 13.2%), where they were watered with 80–100 mL of water every two days.

In June 2018, adults and larvae of *O*. *biannularis* were collected and maintained in plastic cups with perforations to allow the insects to breathe; wetted cotton was placed inside the cups as a source of humidity. The insects fed freely on *I. murucoides* leaves until August 2018, when the herbivory experiments were performed.

#### 4.3.2. Intra- and Interspecific Effect of Herbivory in Stomata of a Receiver Plant

**Figure 5:**
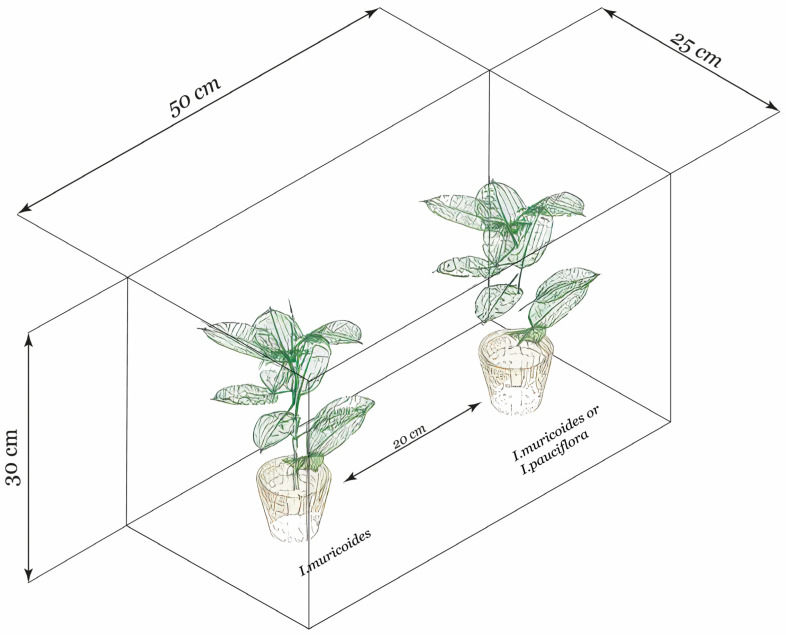
Representative scheme of the confrontation experiment between an emitting plant and a receiving plant. The plants were placed in a closed glass box with a separation of 20 cm. The emitting plants received one of the three following treatments: MeJA (0.5 µM), herbivory by *O. biannularis,* and without herbivory.

Two *Ipomoea* plants (separated by a distance of 20 cm) were put inside each of 18 glass boxes (width 25 cm × length 50 cm × height 30 cm) (Figure 5). In each glass box, one *I. murucoides* plant (putative emitter, from here on designated an emitter) was raffled to one of three treatments: (a) herbivory (H) by *O. biannularis*, (b) without herbivory (WH), and (c) MeJA; thus, there were six boxes per treatment. Half of these six boxes had one *I. murucoides* plant as a putative receiver (intraspecific), and the other three boxes had an *I. pauciflora* plant (interspecific) (Figure 5). The herbivory treatment involved exposing each emitter plant (always *I. murucoides*) to ten chrysomelid larvae/48 h. The emitter plants were covered with a mesh bag to prevent larval migration between plants. The MeJA treatment was reached by sprinkling MeJA (0.5 µM) six times on the emitter plants, three times at the beginning of the experiment, and three times at the 24 h timepoint. The emitters were sprinkled outside the glass box to prevent MeJA from reaching the neighboring receiver plant. This treatment was a positive control because MeJA participates in the defense mechanism against herbivores [71]. The third treatment was the control, where the emitters were sprinkled with distilled water. After the experimental treatments (MeJA, natural herbivory, and control), we waited until new leaves matured in the receiver plant.

Three new leaves were chosen randomly from the middle part of the receiver plant. According to previous observations, leaves of *I. murucoides* and *I. pauciflora* are mainly hypostomatic; thus, we made abaxial impressions of 0.5 cm^2^ between 7:00–8:00 h. A clear nail polish (Bissú^®^) was used to obtain the leaf impressions, which were observed with a confocal scanning laser microscopy (Carl Zeiss, LSM800, Oberkochen, Germany) at the Centro de Desarrollo de Productos Bioticos (Instituto Politecnico Nacional). The samples were mounted on a glass coverslip, and the laser wavelength used was 488 nm at 5% excitation. Focal slices of each sample were made with a pinhole aperture of 1.0 Airy units. All the micrographs were captured with a ESID detector at 200 X using a Plan Neofluar 20X/0.5 objective (Carl Zeiss, Oberkochen, Germany) and all images were stored in the TIFF format with a resolution of 1024 × 1024 pixels. The measured variables were the number of stomata/0.36 mm^2^ and the proportion of open stomata. Fifty-four images were analyzed.

### 4.4. Statistical Analysis

All analyses were carried out in r version 3.6.4 [72], with the libraries ade4 (PCA [73]), ggplot2 (graphics, [74]), ggsci (graphics, [75]), lm4 (mixed linearized models, [76]), and multicomp (multiple comparisons procedures) [77]. For both years, to determine if there is a grouping of herbivory treatments based on EOs and if this correlates with environmental variables (temperature, precipitation, and solar radiation), a principal component analysis (PCA) was performed [78]. The PCA was based on a correlation matrix (r = −1 to 1), and the average area of essential oils that were grouped when their time retention was ±0.2. The significant variables related to each PC were those with r > $\left|0.7\right|$. 

To determine the effect of herbivory on the stomata number of receiver plants (*I. murucoides*, *I. pauciflora*), a generalized mixed linear model with a Poisson error and log link function was performed [79]. The factors were the herbivory treatment (WH, H, and MeJA), the receiver species (fixed effects), the identity of the plant (random effect), and the leaves inside the plants (random effect). On the other hand, to analyze the impact of these factors on the number of open stomata, a generalized linear mixed model was performed for binomial data using the logit link function [80]. Tukey’s multiple comparisons tests were run in case of significant differences from all previous analyses (*p* < 0.05).

## Figures and Tables

**Table 1:** Chemical compounds (VOCs) found in leaves of 2016 and 2017 correlated significantly with two principal components (PC). Numbers in black are significant correlations ($r\geq \left|0.7\right|$). WH = without herbivory, H < 20% = herbivory < 20%, H > 20% = herbivory > 20%, and MD = mechanical damage. The check symbol indicates the presence of VOCs in herbivory treatment. UC means unknown compound. RI means retention index.

Chemical Compound	RI	RI [Reference]	PC1	PC2	2016				2017			
					WH	H <20%	H >20%	MD	WH	H <20%	H >20%	MD
UC (PM = 100.1)	914.50,	−	0.96	0.24					✔	✔	✔	✔
UC (PM = 100.1)	919.23	−	0.96	0.24					✔	✔	✔	✔
UC (PM = 100.1)	922.54	−	0.96	0.25					✔	✔	✔	✔
(Z)-2-Hexenal	980.70	848 [26]	−0.24	0.79		✔	✔	✔		✔	✔	✔
(*Z*)-3-Hexen-1-ol	984.00	852 [27]	0.20	−0.80	✔				✔			
UC (PM = 189.2)	1454.54	−	0.96	0.24					✔	✔	✔	✔
UC (PM = 189.2)	1466.32	−	0.81	0.12					✔	✔	✔	
UC (PM = 189.2)	1468.09	−	0.70	−0.37					✔	✔		
α-Cubebene	1486.98	1343 [28]	−0.76	0.04		✔	✔	✔			✔	
UC (PM = 204.2)	1518.08	−	0.96	0.24					✔	✔	✔	✔
UC (PM = 204.2)	1547.63	−	0.96	0.24					✔	✔	✔	✔
γ-Cadinene	1580.85	1513 [29]	0.22	−0.80	✔				✔			
α-Caryophyllene	1593.37	1455 [30]	−0.96	−0.24	✔	✔	✔	✔				
UC (PM = 189.2)	1613.50	−	0.70	−0.37					✔	✔		
UC (PM = 189.2)	1616.12	−	0.70	−0.37					✔	✔		
UC (PM = 204.2)	1634.81	−	0.70	−0.37					✔	✔		
Germacrene D	1645.82	1486 [31]	0.08	−0.70	✔	✔		✔	✔	✔		✔
γ-Elemene	1649.66	1435 [32]	−0.22	0.80		✔	✔	✔		✔	✔	✔
α-Farnesene	1661.37	1511 [33]	−0.73	−0.43	✔	✔		✔				
Cadina-1,4-diene	1676.74	1843 [34]	−0.76	0.04		✔	✔	✔				
α-Muurolene	1683.55	1499 [35]	0.81	−0.35	✔	✔	✔	✔	✔	✔	✔	✔
Nerolidol	1710.41	1565 [36]	−0.08	0.70			✔				✔	
7(11)-Selinen-4α-ol	1757.47	1693 [37]	−0.15	−0.82	✔	✔	✔	✔	✔	✔	✔	✔
1(10),5-Germacradien-4-ol	1763.32	1567 [38]	−0.96	−0.24	✔	✔	✔	✔				
τ-Cadinol	1783.28	1649.8 [29]	0.05	0.80			✔	✔		✔	✔	✔
τ-Muurolol	1786.94	1660 [31]	0.15	−0.82	✔	✔	✔	✔	✔	✔	✔	✔
δ-Cadinol	1790.42	1646 [39]	−0.96	−0.24	✔	✔	✔	✔				
Patchouli alcohol	1832.19	1666 [40]	−0.96	−0.24	✔	✔	✔	✔				
2(*Z*),6(*Z*)-Farnesol	1871.54	1698 [41]	−0.96	−0.24	✔	✔	✔	✔				
Phytol	2267.10	2119 [42]	−0.94	−0.30	✔	✔	✔	✔	✔	✔	✔	✔

## Data Availability

The original contributions presented in this study are included in the article/Supplementary Material. Further inquiries can be directed to the corresponding author.

## Supplementary Materials

The following supporting information can be downloaded at: https://www.mdpi.com/article/10.3390/plants13223124/s1, Figure S1: Chromatograms of treatments of herbivory in two years (2016 and 2017), (a) Without herbivory 2016, (b) Herbivory < 20% 2016, (c) Herbivory > 20% 2016, (d) Mechanical damage 2016, (e) Without herbivory 2017, (f) Herbivory < 20% 2017, (g) Herbivory > 20% 2017, (h) Mechanical damage 2017. Figure S2: Spectra of Chemical compounds of *Ipomoea murucoides*, (a) Without herbivory: (Z)-3-Hexen-1-ol, (b) Without herbivory: γ-Cadinene, (c) Herbivory > 20 %: nerolidol.
