# Supplementary material for: Biennial Variation and Herbivory Affect Essential Oils of Ipomoea murucoides and Stomata Density of Neighbor Plants
Source: Plants (Basel). 2024 Nov 6;13(22):3124. doi: 10.3390/plants13223124 (PMC11597795; doi:10.3390/plants13223124)

SUPPLEMENTARY MATERIAL.

Figure S1: Chromatograms of treatments of herbivory in two years (2016 and 2017)

(a) Without herbivory 2016

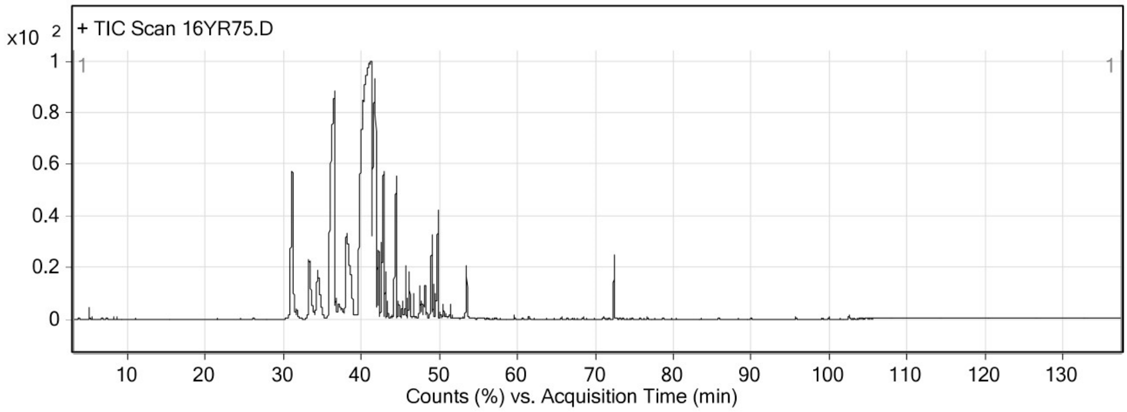

(b) Herbivory < 20% 2016

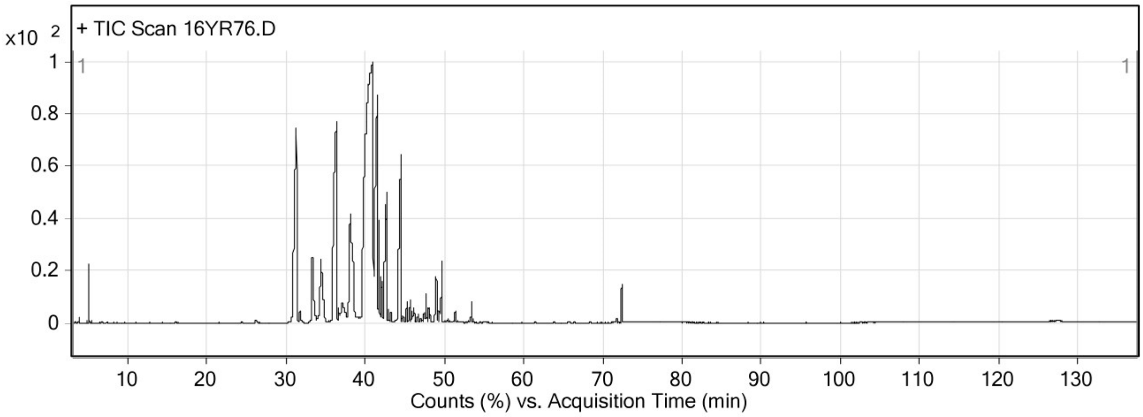

(c) Herbivory > 20% 2016

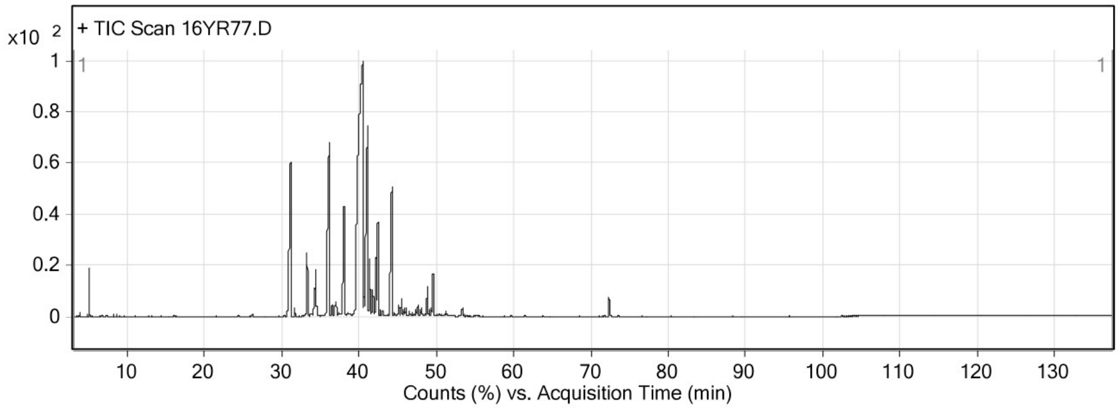

(d) Mechanical damage 2016

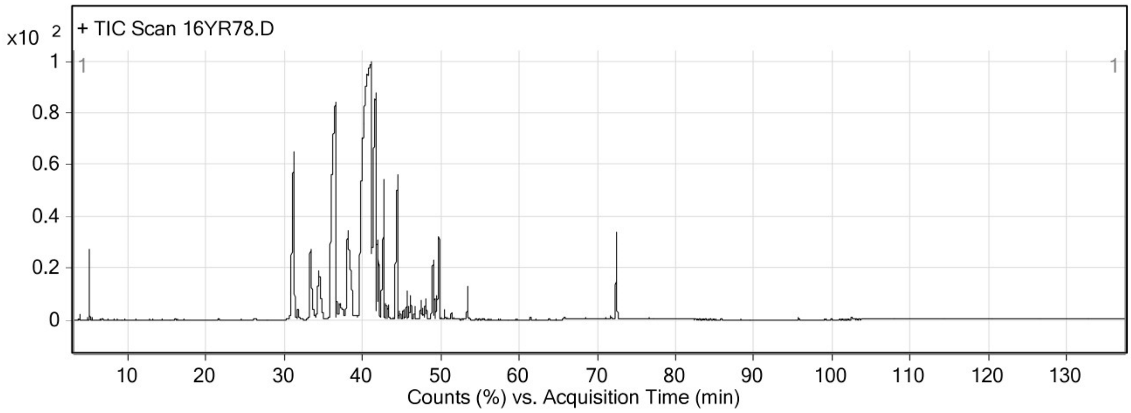

(e) Without herbivory 2017

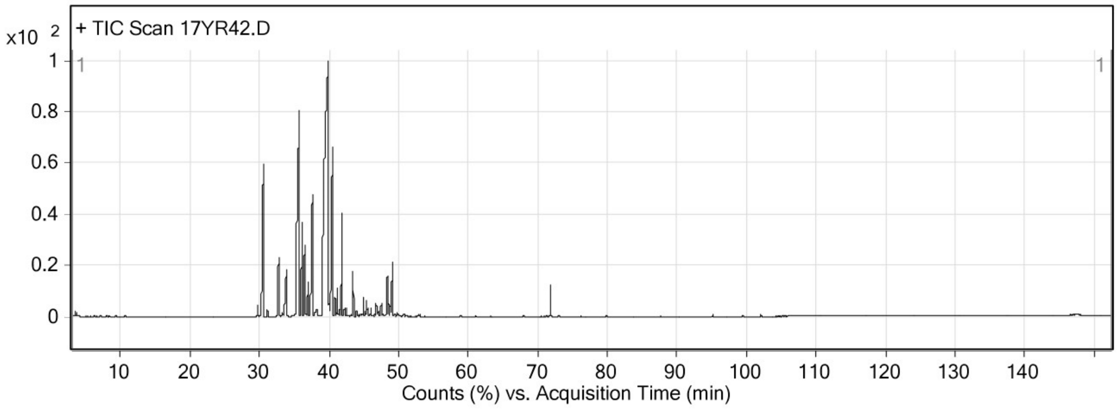

(f) Herbivory < 20% 2017

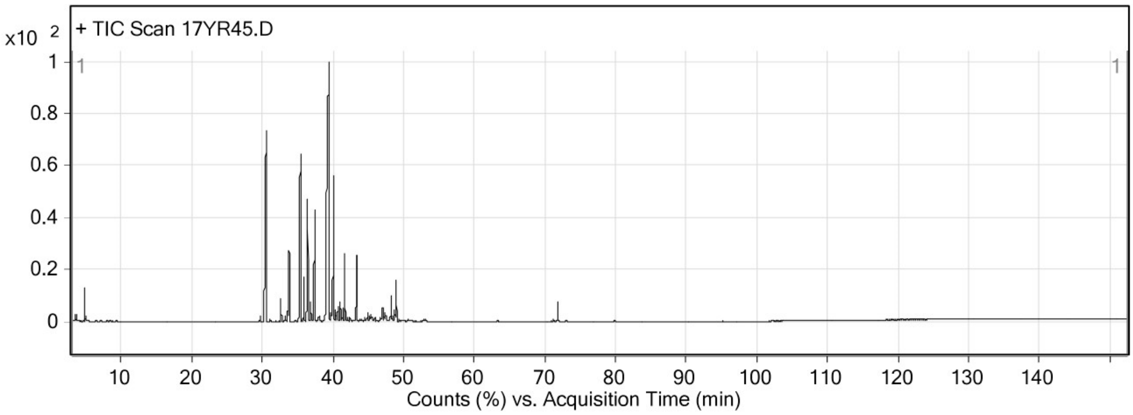

(g) Herbivory > 20% 2017

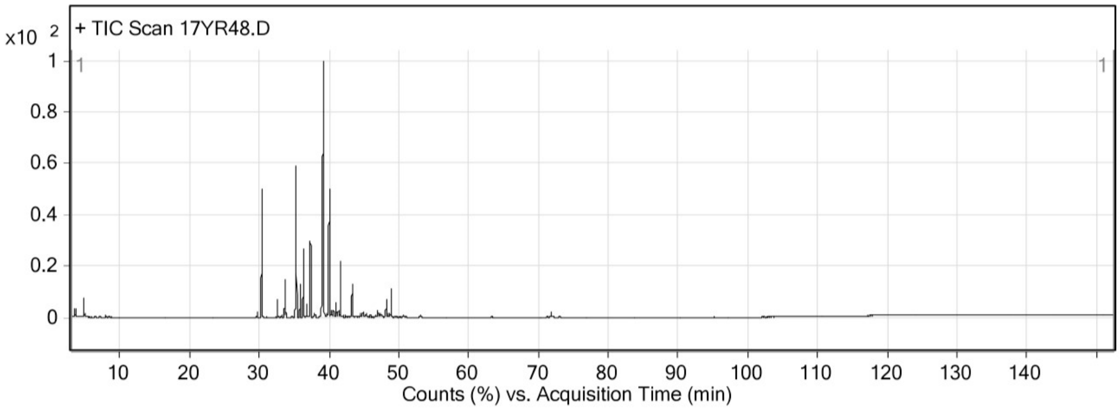

(h) Mechanical damage 2017

Figure S2. Spectra of chemical compounds of *Ipomoea murucoides*.

(a) Without herbivory: (Z)-3-Hexen-1-ol

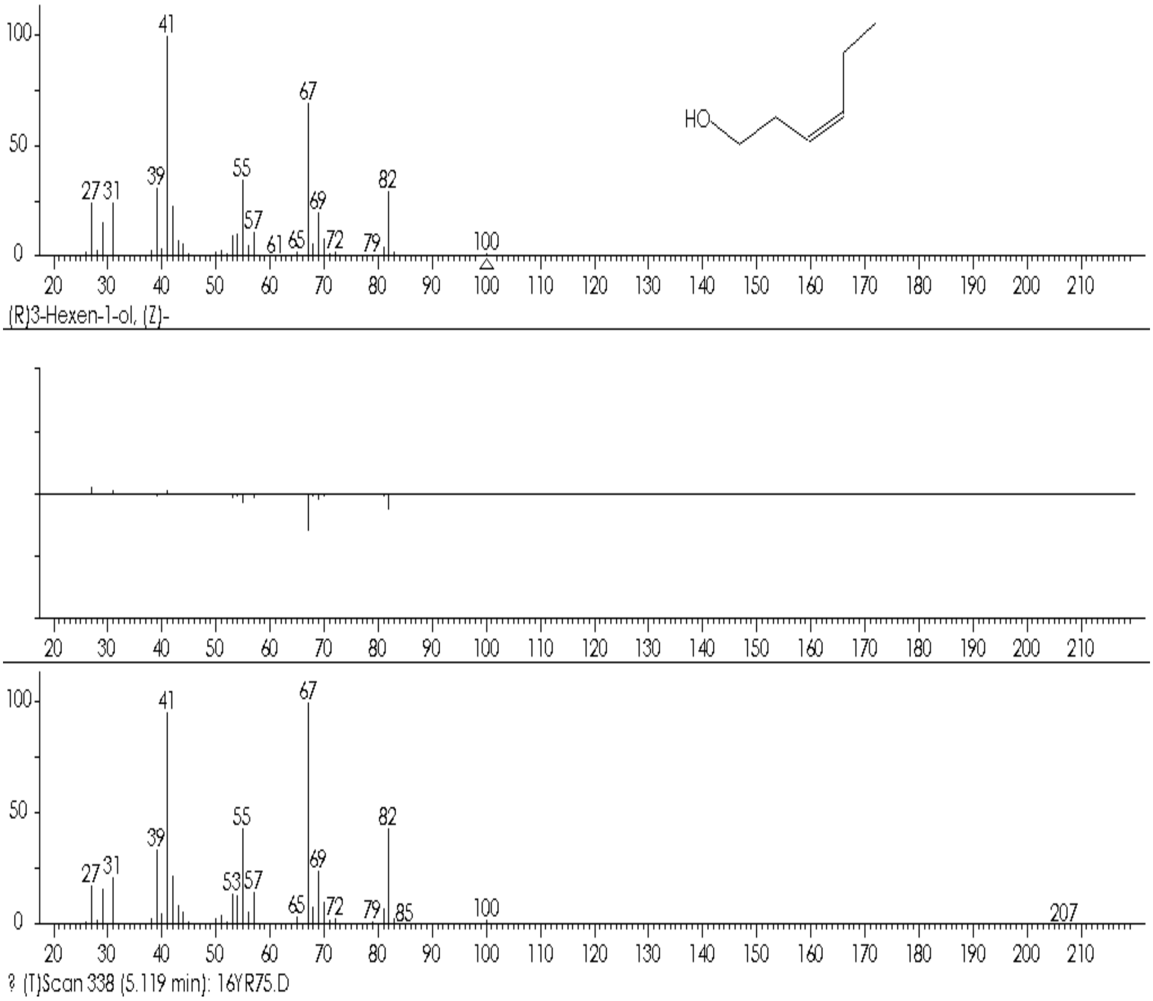

(b) Without herbivory: γ-Cadinene (https://webbook.nist.gov/)

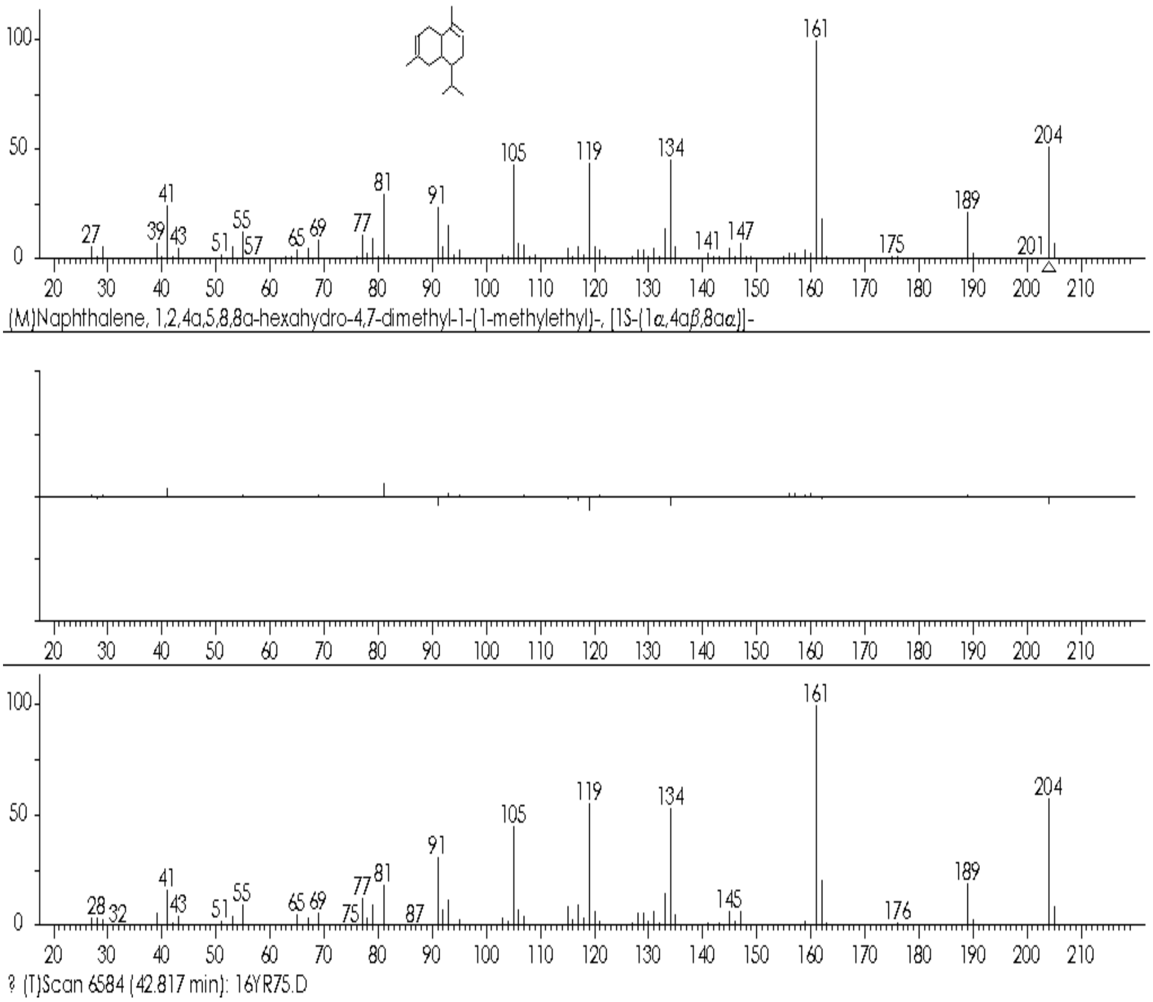

(c) Herbivory > 20 %: nerolidol Pub-Chem (https://pubchem.ncbi.nlm.nih.gov/substance/249929331#section=2D-Structure)

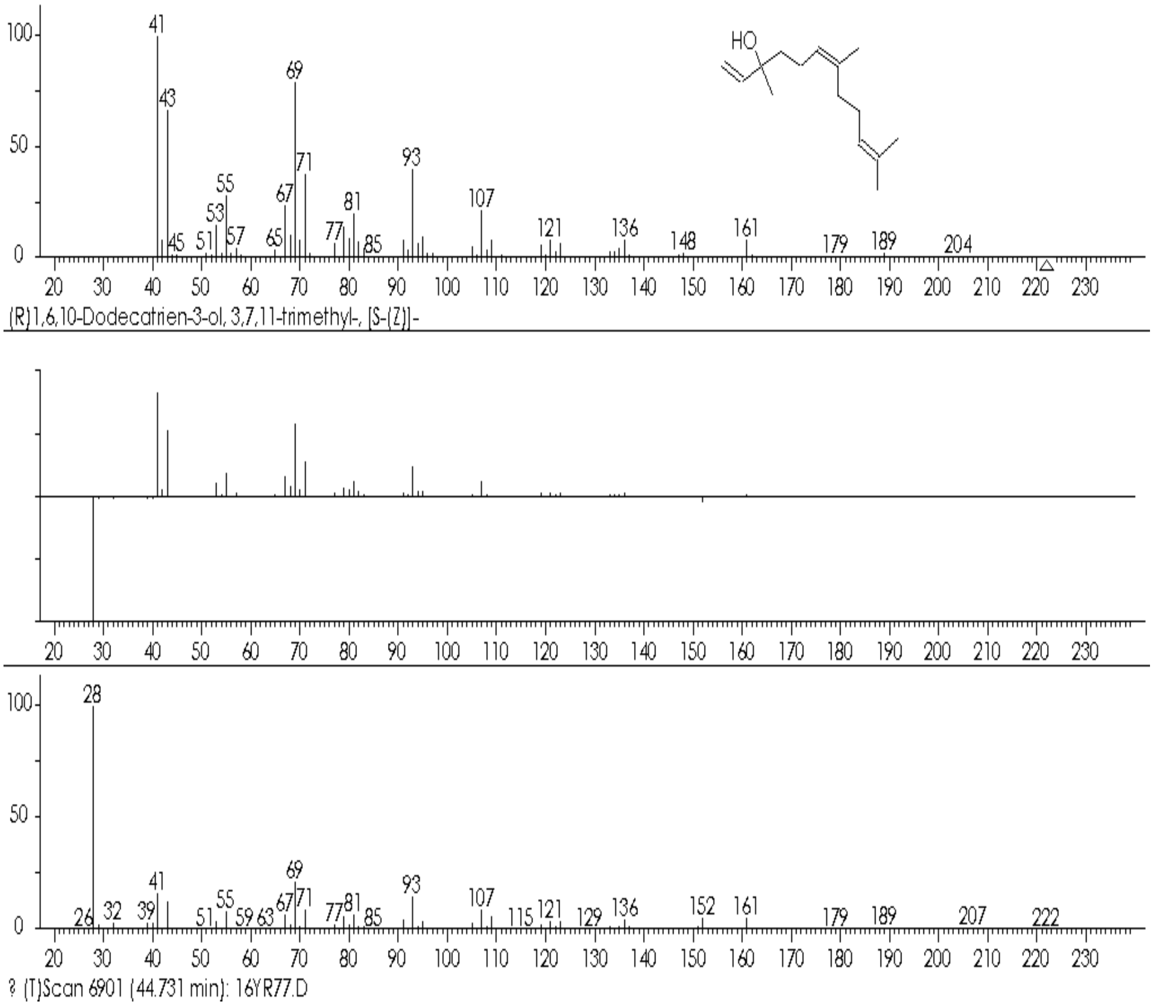

Supplement: Supplementary file 1 [file plants-13-03124-s001.zip › plants-3244851-supplementary.pdf]
